# Two Cases of Poisoning with Illuminating Gas Successfully Treated by the Inhalation of Oxygen*Related at a meeting of the New York Medical and Surgical Society, February 24, 1883.

**Published:** 1883-12

**Authors:** Alonzo Clark

**Affiliations:** Emeritus Professor of Pathology and Practical Medicine in the College of Physicians and Surgeons


TWO CASES OF POISONING WITH ILLUMINATING GAS SUCCESSFULLY TREATED BY THE INHALATION OF OXYGEN.* BY ALONZO CLARK, M.D., LL. D., Emeritus Professor of Pathology and Practical Medicine in the College of Physicians and Surgeons.

So far as I know, these two cases of poisoning with illuminating gas are the only cases of this sort on record in which the treatment by the inhalation of oxygen has been resorted to. The histories of the cases, somewhat condensed from the written reports of the house physicians, are as follows:

CASE I.—A woman, forty years old, was brought to Bellevue Hospital and admitted into my service May 30, 1882. She and her daughter had gone to bed the night before, and about noon they were found in a state of unconsciousness, the room where they had been lying for fifteen hours being filled with illuminating gas, the odor of which they had noticed on going to bed. On recovering from her insensibility, the patient could remember nothing that had happend from the time of her going to bed until she regained consciousness in the hospital ward, after the lapse of 24 hours.

* Related at a meeting of the New York Medical and Surgical Society, February 24, 1883.

At the time of her admission, she was found to be suffering from pulmonary œdema; the radical pulse was scarcely perceptable, she was unconscious and cyanotic, her extremities were cold, there was trismus with rigidity of the flexor muscles, the urine was passed involuntarily, the pupils were slightly contracted, and a frothy mucus issued from the mouth. Her temperature was 96·5°F., and her respiration 40.

I entered the hospital shortly after she was brought in, and directed the inhalation of oxygen. Its administration was kept up for three hours. In addition, dry cups were applied over the chest, and tincture of digitalis was given endermically—in all, to the amount of thirty minims. Whisky also was given subcutaneously, and hot-water bottles were applied to the extremeties. Occasionally, too, the patient was aoused by flagellation.

The treatment extended over a period of four hours, at the end of which time the woman began to show signs of returning consciousness; the pulse became more perceptable and regular, warmth returned to the extremities, and the temperature and the respiration were found to be normal. The next day the patient was able to tell her own story, and was soon afterwards discharged.

CASE II.—The daughter of the first patient, a girl twelve years old, was admitted at the same time, in Dr. Loomis' service. Her breath had a very strong odor of gas; she was com-completely comatose, the breathing being stertorous, the eyes closed, the conjunctivæ insensitive, and the pupils contracted and insensible to light; the pulse was 156, extremely feeble, and occasionally intermitting; the respirations were 30 and shallow. There were clonic spasms involving the left side of the body, with bird claw contraction of the fingers of both hands. The urine and fæces were passed involuntarily. The face and clothing showed evidence that the patient had vomited a good deal before reaching the hospital, but after her admission there was no vomiting.

Seeing that my patient was being treated by the inhalation, the house physician, Dr. Pryor, resorted to the same treatment in this case, and, in addition, gave a hypodermic injection of a sixtieth of a grain of sulphate of atropine. This patient also recovered.—*New York Medical Journal.*

